# Epileptic spikes detector in pediatric EEG based on matched filters and neural networks

**DOI:** 10.1186/s40708-020-00106-0

**Published:** 2020-05-24

**Authors:** Maritza Mera-Gaona, Diego M. López, Rubiel Vargas-Canas, María Miño

**Affiliations:** University of Cauca, Popayán, Colombia

**Keywords:** Matched filter, Spike detection, Epilepsy, Seizure, Neural networks

## Abstract

**Abstract:**

The electroencephalogram (EEG) is a tool for diagnosing epilepsy; by analyzing it, neurologists can identify alterations in brain activity associated with epilepsy. However, this task is not always easy to perform because of the duration of the EEG or the subjectivity of the specialist in detecting alterations.

**Aim:**

To propose the use of an epileptic spike detector based on a matched filter and a neural network for supporting the diagnosis of epilepsy through a tool capable of automatically detecting spikes in pediatric EEGs.

**Results:**

Automatic detection of spikes from an EEG waveform involved the creation of an epileptic spike template. The template was used in order to detect spikes by using a matched filter, and each spike detected was confirmed by a Neural Network to improve sensitivity and specificity. Thus, the detector developed achieved a sensitivity of 99.96% which is better than the range of what has been reported in the literature (82.68% and 94.4%), and a specificity of 99.26%, improving the specificity found in the best-reviewed studies.

**Conclusions:**

Considering the results obtained in the evaluation, the solution becomes a promising alternative to support the automatic identification of epileptic spikes by neurologists.

## Introduction

Reading EEGs by specialists is a task that consumes considerable effort and time due to the duration of EEG signal recordings. In general, EEG records have durations ranging from 20 to 30 min and in some cases the records are even longer (48 or 72 h), representing one of the main causes of the high cost of diagnosing neurological diseases such as epilepsy [1]. Similarly, the difficulty of diagnosing this kind of disease increases in developing countries, due to the lack of medical personnel. In countries such as Colombia, for example, there is a rate of one neurologist per 200,000 inhabitants [2]; therefore, it is difficult to guarantee diagnosis and timely attention to patients. This situation is more worrisome in the case of patients residing in rural areas because specialists are located in the clinical centers of the main cities.

Considering the above, automatic detection of different abnormal events presented in EEG signals arises as an alternative to reduce the time involved in reading an EEG signal and to increase the opportunity of EEG reading services, because once the abnormalities on the signal are identified, the specialist would only have to confirm or reject them.

The automatic analysis of EEGs is a research field where different approaches have been developed in order to offer tools that facilitate the reading of EEG records, especially for those of long duration. In [3], the authors proposed to classify epileptiform events using time–frequency analysis and a random forest-based classifier, achieving an accuracy of 83%. Likewise, in [4] the authors used features extracted from wavelet coefficients to classify EEG segments^1^ with a 93% sensitivity and specificity. In [5], the research team developed a tool based on neural networks for the detection of epileptic seizures, obtaining an accuracy, specificity, and sensitivity of 88.67%, 90% and 95%, respectively.

Considering the above, we can note that the main challenge in future solutions is to improve the percentages of effectiveness and reliability of the detection or classification of epileptic seizures. In previous works, the authors used a general model to classify or detect different patterns of epileptic discharges. However, this could reduce the effectiveness of detection or classification because the epileptic abnormalities are difficult to represent under a unique model. Consequently, in order to increase the reliability of the EEG reading, some researches have implemented solutions for identifying specific patterns instead of classifying all kinds of abnormalities.

The aim of this paper is to present an epileptic spike detector based on matched filter and neural networks for supporting diagnosis of epilepsy through a tool capable of automatically detecting spikes in pediatric EEGs. Automatic detection of spikes from an EEG waveform involves the identification of an epileptic spike template. The template is used to detect similar spikes by using a matched filter. The detection process is divided into two phases: the first one uses a matched filter as a classifier to determine if a small segment of EEG is a spike, and second one uses a classifier based on Neural Networks to confirm if the spike detected in the previous phase is actually an abnormality or a false positive. This strategy seeks to reduce the number of false positives and improve the sensitivity and specificity of the final detector.

The rest of this paper is organized as follows: section 2 describes the dataset used for supporting the development and evaluation of the proposal, the theoretical description of the Matched Filter, Neural Networks and the development of the detector for the automatic identification of epileptic spikes. Section 3 presents the experimental evaluation of the sensitivity and specificity of the epileptic spike detector. Section 4 describes the discussion of the results and main contributions. Finally, section 5 describes the conclusions of this work.

## Materials and methods

This section presents a description of the main materials, methods and concepts considered for the implementation of the automatic detection of epileptic spikes in an EEG signal.

### Database

In this research, we collected 200 electroencephalograms from children with suspected epilepsy. This collection was made as part of the Neuromotic project, which had as its general objective to develop a TeleEEG system to support the diagnosis of epilepsy in rural areas in Colombia [6]. As part of this project, we developed a component to support the reading of EEGs by a neurology professional.

In the construction of the dataset and in accordance with bioethics standards, we obtained an informed consent for each EEG record. Such consent was approved by the Ethics Committee of University of Cauca, Colombia. Each EEG record was acquired using the BWII EEG device and the BW Analysis software, both developed by Neurovirtual. The device has FDA certification.

Each EEG record was acquired under the electrode positioning system 10–20 [7], considering a sampling rate of 200 samples per second, and an approximate duration of 30 min. Some EEG records were taken in patients in the waking state (46 records) and others in sleep (54 records). All patients were asked to fall asleep during the recording of the EEG to decrease the appearance of artifacts in the signal; however, not all of them were able to fall asleep.

Once the records were digitized, we conducted an annotation process with the help of a neuropediatrician. The process included a review of EEGs performed by the neuropediatrician to identify the segments where epileptic alterations occurred. We documented the beginnings and ends of all epileptic abnormalities identified by the doctor. As a result of the annotation process, we built a dataset with abnormal and normal segments extracted from EEG records. The dataset is available on GitHub.^2^

### Matched filter

**Fig. 1:**
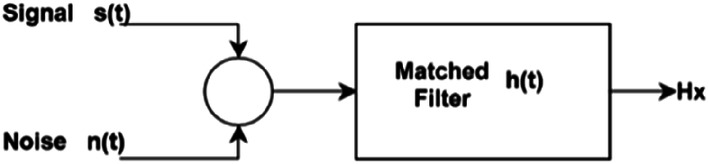
Detection scheme Source: adapted from [8]

Matched filters are basic signal analysis tools used to extract known waveforms from a signal that has been contaminated with noise [8]. The model used for the extraction or detection of the wave can be seen in Fig. 1.

The diagram defined in Fig. 1 describes the implementation of a filter *h(t)* to extract the signal *s(t)* contaminated with noise *n(t)*. Hypothesis $H_{x}$ is obtained as a result of applying *h(t)*. In this diagram, the null (*H*_*0*_) and alternative (*H*_*1*_) hypotheses are considered in Eqs. (1, 2). If the waveform sought is present in the signal, hypothesis *H*_*1*_ is confirmed. Otherwise, hypothesis *H*_*0*_ is confirmed. In the context of the detection of epileptic spikes, *x(t)* is a function to describe the brain activity (EEG); noise *n(t)* represents the normal brain activity of a patient (EEG base rhythm), signal *s(t)* the epileptic spike to be found, *H*_*0*_ normal activity of the patient, and *H*_*1*_ the presence of the epileptic spike:

1 $$H_{0} :x\left( t \right) = n\left( t \right),$$

2 $$H_{1} :x\left( t \right) = s\left( t \right) + n\left( t \right).$$

This mechanism works very well in practice when we seek a known pattern or waveform, because the filter allows to maximize the SNR (signal-to-noise ratio) of the filtered signal and to reduce the effect of noise on the original signal [9]. However, when waveforms are not known, the method does not work efficiently.

**Fig. 2:**
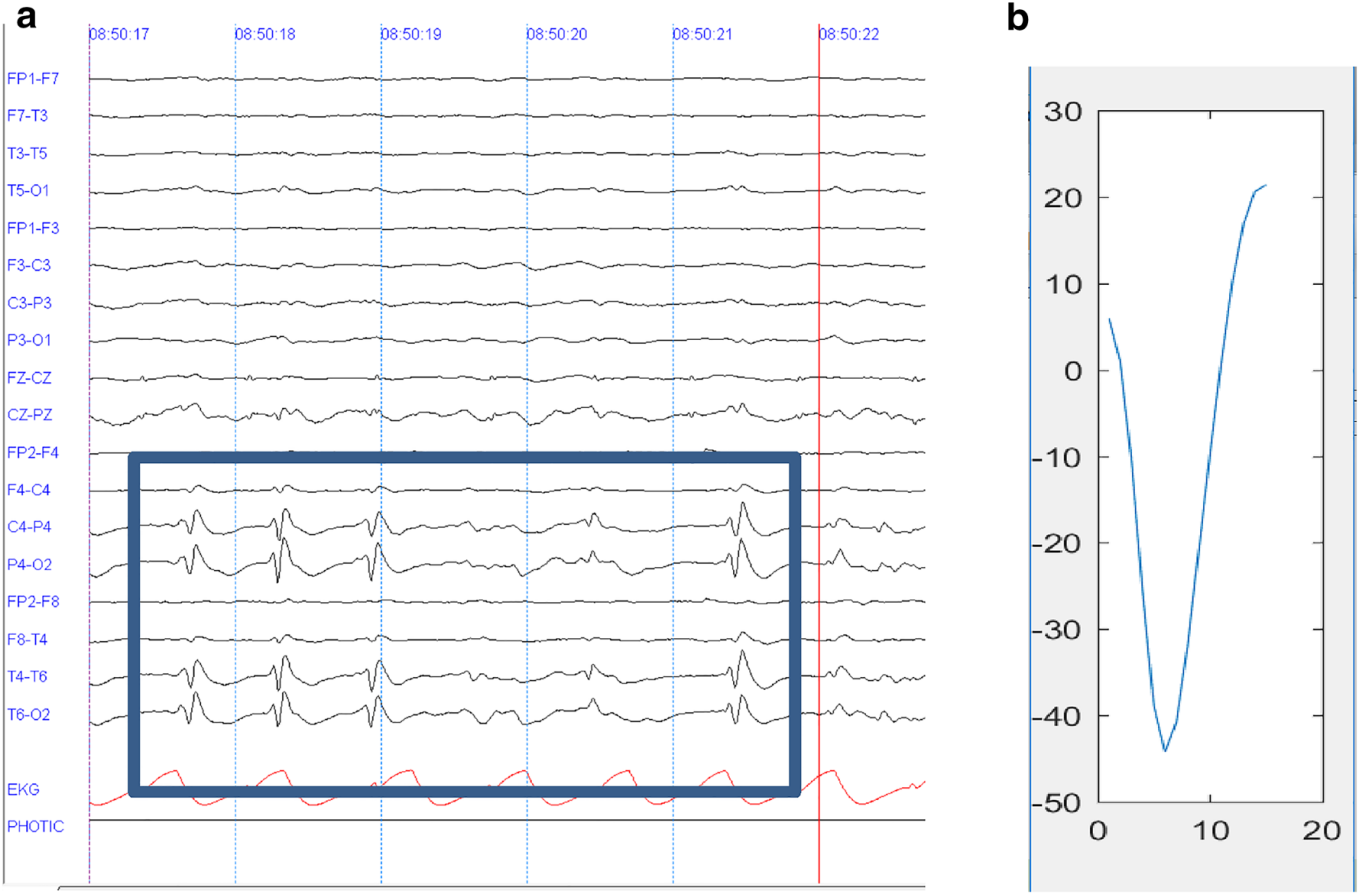
Visual inspection of spikes. **a** Epileptic spikes in the base rhythm. **b** Epileptic spike pattern

In this work, the main goal is to develop a tool that supports the diagnosis of epilepsy through the identification of epileptiform events. For this purpose, we identified characteristic patterns that describe the presence of an epileptic discharge. In this sense, it could be observed that epileptic seizures generate electric shocks on some areas of the brain generating unexpected changes in the waveform of EEGs. In some cases, the appearance of waveforms is identified periodically or semiperiodically or simply by the disorganization of the brain electrical activity of the patient. Some of the most desired patterns by neurologists during the inspection of EEGs correspond to spikes (both narrow and broad). Thus, we propose to build a tool to detect spikes automatically by using a pattern that works as a reference template. This template was constructed by averaging 25 segments diagnosed as spikes by a neuropediatric expert in reading EEGs. Figure 2a shows an example of the appearance of epileptic spikes in the base rhythm of the EEG wave on channels 17, 18, 22 and 23 of the EEG.

The epileptic pattern of Fig. 2b was constructed using epileptic spikes from 10 patients. Considering the visual analysis performed by the neurologist, it defined that the size of the segments of epileptic spikes extracted should contain data of 15 samples, 13.33 ms, in order to capture the data from the beginning of the spike to the end of it.

### Epileptic spike detector based on a matched filter

**Fig. 3:**
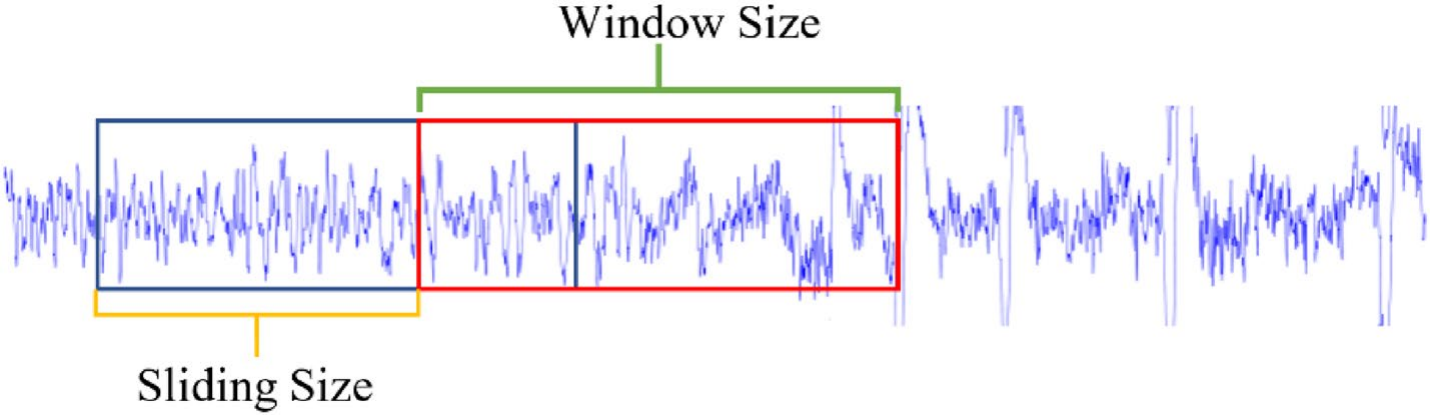
Analysis scheme by window

Considering the wave pattern that describes an epileptic spike, we constructed a spike detector algorithm using a matched filter and sliding windows over an EEG channel. The algorithm is defined below: 
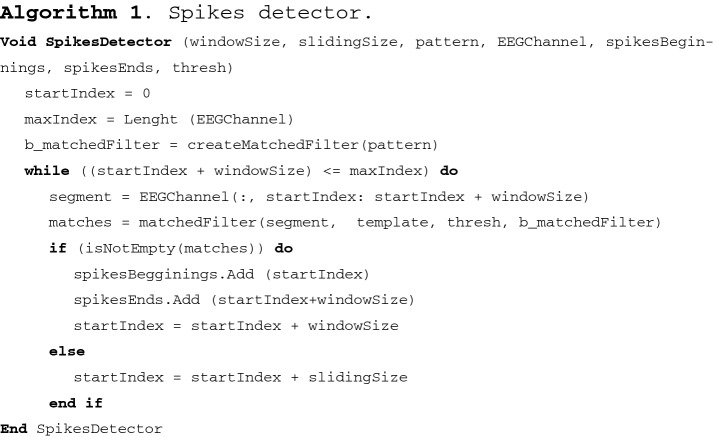


The algorithm receives 7 arguments, size of window, size of sliding, pattern, EEG channel, beginnings and ends of segments detected, and threshold. The size of the window allows the identification of the beginning and end of the segment to be analyzed, while the size of the sliding allows us to know how many samples move to the right of the beginning of the segment that has been analyzed. The threshold establishes the minimum level of similarity, between the analyzed window and the spikes template, to be considered as a spike. Figure 3 illustrates the aforementioned process.

The *pattern* corresponds to the template constructed from the epileptic spikes, *EEGChannel* corresponds to a channel extracted from the EEG in which the pattern will be searched. *spikesBeginnings* and *spikesEnds* correspond to the arrangements which store the beginnings and ends of the segments that have presence of the pattern of epileptic spikes, and the function *createMatchedFilter* creates a matched filter based on the template. The Spikes Detector algorithm analyzes the entire EEG channel, extracting segments through the sliding window. With the implementation of the Matched Filter (Algorithm 2), each window extracted is checked to determine if in that window there is an epileptic spike pattern.

The algorithm that describes the Matched Filter is described below: 
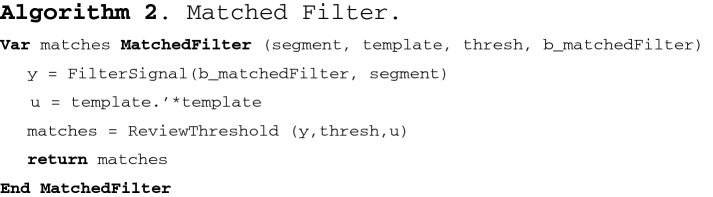


In the above algorithm, *segment* describes the segment to be evaluated, *template* represents the epileptic spike pattern, *thresh* sets a detection threshold, which was established empirically in 0.7 by testing values between 0.6 and 1; *b_matchedFilter* contains the matched filter based on the template, *y* contains the segment filtered with the matched filter, *u* stores the autocorrelation matrix of the template, and function *ReviewThreshold* establishes if *y* exceeds the threshold. The autocorrelation matrix allows us to detect the presence of patterns in a signal; in this case, the autocorrelation matrix was used for detecting the pattern of spikes in the brain activity.

### Backpropagation neural networks

**Fig. 4:**
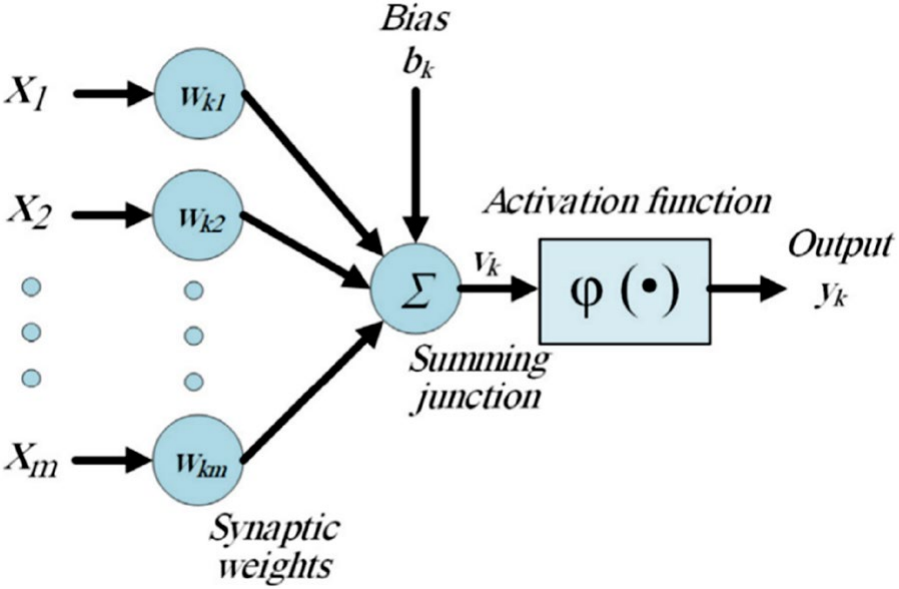
Neural Network architecture Source: adapted from [16]

The basic unit of a neural network is a neuron and has the function of receiving inputs, processing them, and producing one output. Thus, a Neural Network is defined as a combination of a set of connected neurons. According to the basic architecture described in Fig. 4, the links that connect the neurons are called synapses and have a weight $w_{kj}$, which are used in a hidden layer by the activation function to calculate one output [16].

Backpropagation neural networks are described by a hierarchical architecture where one input layer, several hidden layers and one input layer are connected [17]. The training of neural network is conducted following a backpropagation scheme. Thus, the training process is divided into three steps: (i) the network makes a conjecture, (ii) this is measured by a loss function, and (iii) the error is backpropagated to adjust the network.

## Results

**Fig. 5:**
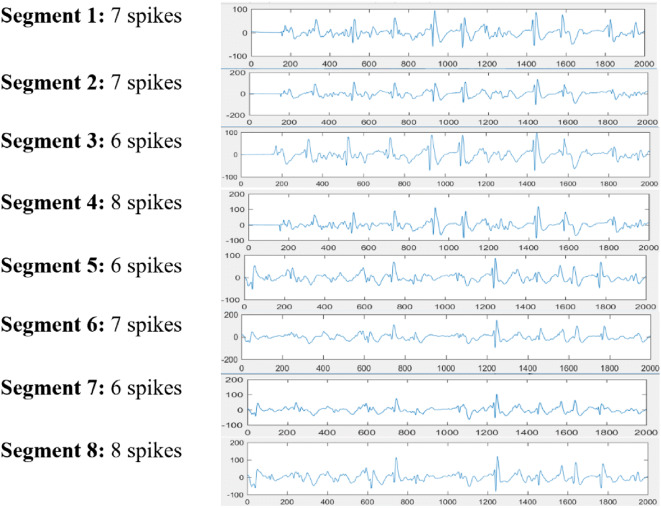
Description of each segment

The final evaluation used recordings of brain activity of 108 patients. Such records were divided into 2 groups: (i) EEGs of 8 patients with spikes in their brain activities and (ii) EEGs of 100 patients without spikes in their brain activities. We extracted 8 test segments from EEGs of the first group (the only patients in the collection with this kind of abnormality that were not considered in the construction of the Matched Filter). Considering the annotations made on the dataset and segments selected, we know the beginnings and ends of 56 epileptic discharges that occur in the form of spikes. In this sense, the spike detector was used for each test segment and the correctly identified, badly identified, and unidentified spikes were counted in Table 2 to determine the sensitivity and specificity of the detection. Figure 5 describes examples of spikes (epilepsy episodes) contained in the extracted segment with abnormalities.

Each segment described in Fig. 5 was reviewed by the spike detector. The results can be seen in Table 1.

**Table 1:** Results of the evaluation

Segment	Real spikes	Spikes detected
S1	7	25
S2	8	23
S3	6	20
S4	8	31
S5	6	6
S6	7	25
S7	6	15
S8	8	22

In the results obtained, the number of spikes detected in each test segment is greater than the actual number of spikes. With this in mind, each spike identified by the detector was reviewed to analyze the reason of the error. It was possible to identify that, in some cases, the detector was identifying a real spike twice or three times, due to the reduced size of the sliding window and, in other cases, the detector also considered the abrupt fall of the slow waves that occur just after the spike occurrence. It is also important to mention that the neurologists annotating the EEG also considered slow waves an abnormality. Thus, the spikes detected with close beginnings (difference between beginnings less than 20 samples) were considered as a single one because these spikes are multiple windows generated by sliding windows from a single spike or a spike with a slow wave.

Considering the above, Table 2 presents the results of the evaluation eliminating repeated spikes, the detection of slow waves and the number of spikes not detected.

**Table 2:** Results of the evaluation using threshold 0.7

Segment	Real spikes	Detected spikes	Slow waves	Spikes not detected	Wrongly detected spikes
S1	7	7	8	0	6
S2	8	8	5	0	4
S3	6	6	6	0	2
S4	8	8	7	0	5
S5	6	5	4	1	2
S6	7	7	8	0	3
S7	6	6	6	0	4
S8	8	8	7	0	5
Total	56	55	51	1	31

According to the results obtained in Table 2, the built-in matched filter achieved a sensitivity of 98.28% using a threshold of 0.7. To calculate the sensitivity, we used data of 8 patients that presented spikes in their brain activity. For the evaluation, we extracted 8 segments (the size of extracted segments is 2.000 samples) where spikes appear, and the inspection of these segments was conducted by decomposing each segment by sliding window. In this manner, we generated for each segment of 2000 samples a total of 369 windows, which were evaluated by using the matched filter. Whereas there were 8 segments, we reviewed 2.952 windows and obtained a specificity of 98.94%. In addition, we extracted the test segments from the brain activity of 8 new patients. That means, the template of matched filter was built with data of a group of patients and evaluation with data of a different group.

Considering that, in a Matched Filter, the threshold establishes how similar the template and the segments are. We tested different thresholds in order to determine an appropriate threshold. Comparing the results, if the threshold is close to 1 the result is that the specificity increases, and the sensitivity decreases slightly. Table 3 describes the results using a threshold of 0.9. Sensitivity was of 91.07% and specificity of 99.96%.

**Table 3:** Results of evaluation using threshold of 0.9

Segment	Real spikes	Detected spikes	Slow waves	Spikes not detected	Wrongly detected spikes
S1	7	7	1	0	0
S2	8	8	12	0	1
S3	6	5	6	1	0
S4	8	7	6	1	0
S5	6	5	1	2	0
S6	7	7	9	0	0
S7	6	4	2	2	0
S8	8	8	5	0	0
Total	56	51	42	6	1

In order to increase the reliability and robustness of our evaluation, we used data from 100 new test segments (2.000 samples). Because in our dataset of 200 EEGs there are no more patients with spikes, we included test segments from EEGs of 100 patients with non-spike activity. The goal is to evaluate the performance of the matched filter with data from different patients and other types of wave patterns. Given the sliding window used to decompose the test segments into windows, we generated 36.900 windows, which were reviewed one by one by the matched filter. The results of this evaluation showed that matched filter detected 53 false positives and 36,847 true negatives. Because the test data does not contain spikes, the sensitivity could not be calculated, and the specificity was 99.85%.

**Fig. 6:**
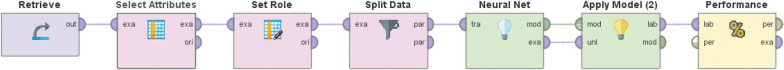
Pipeline of spike detection using a Neural Network

Considering the results of the evaluation of Matched Filter, we implemented a neural network and evaluated according to a similar scheme described in Fig. 6. For the evaluation, we built a dataset with data of 400 spikes and 400 segments with normal brain activity. The segments were transformed according to the feature extractors used in [18]. The neural network was trained with 70% of the data and evaluated with the remaining 30% of the data. The separation of the training and test data was a random process.

The hyperparameters of neural network were fixed empirically by testing different values. Thus, we defined the neural network setting with 500 training cycles, a learning rate of 0.3, a momentum of 0.2 and an epsilon error of 1.0E − 5.

Table 4 describes the confusion matrix with the results of the evaluation of the neural network. According to the results, the sensibility, specificity, and precision were perfect. It is important to mention that although the perfect performance could be considered as suspected, this is the result of reducing the problem of comparing spikes with a multitude of abnormalities to comparing spikes with normal activity.

**Table 4:** Results of the evaluation of the Neural Network

	Spikes	No spikes	Class precision
Pred. spikes	120	0	100.00%
Pred. no spikes	0	120	100.00%
Class recall	100.00%	100.00%	

**Fig. 7:**
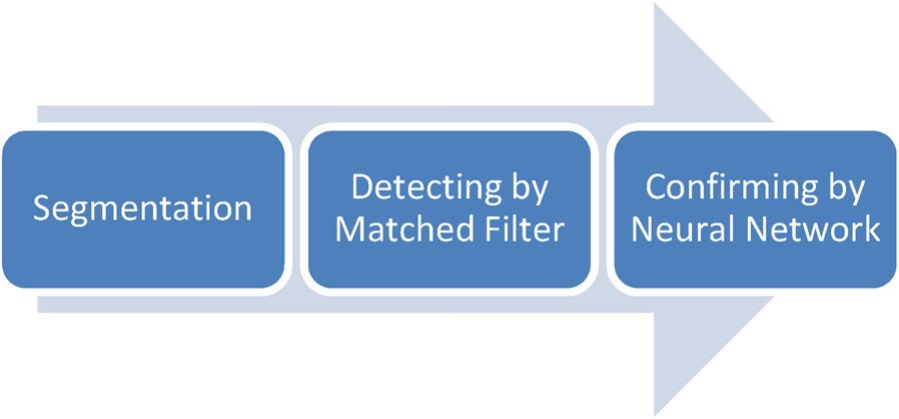
Process of final detection

Once the neural network was trained and validated, we used it to confirm (classify) the spikes detected by the Matched Filter. Figure 7 describes the process used to integrate three steps: (i) the analysis by sliding window, (ii) the detection based on analyzing single segments using a Matched Filter, and (iii) the confirmation of the detections made by the Matched Filter by using the neural network. This approximation allowed us to join the power of Matched Filters to detect segments with high probability of being spikes in signals with spikes, artifacts, noise, and different types of abnormalities, with the capability of classification of Neural Networks to confirm the detections made by Matched Filter.

Table 5 shows the evaluation of the Neural Network to confirm the detected spikes by the matched filter. The precision and sensitivity were of 100% and the specificity was of 99.89%.

**Table 5:** Results of reviewing detected spikes by the neural network

Segment	Real spikes detected by MF	Detected spikes by MF	Confirmation by NN	Spikes not detected	Wrongly detected spikes
S1	7	13	7	0	0
S2	8	11	8	0	2
S3	6	8	6	0	0
S4	7	13	7	0	0
S5	6	7	6	0	1
S6	7	10	7	0	0
S7	6	10	6	0	0
S8	8	13	8	0	0
Total	55	85	55	0	3

Table 6 shows the results of the detection of the built detector. Thus, the precision, sensitivity and specificity reached with the scheme based on a Matched Filter and a Neural Network were of 99.96%, 99.26% and 99.89%, respectively.

**Table 6:** Results of final evaluation

Segment	Real spikes	Detected spikes	Spikes not detected	Wrongly detected spikes
S1	7	7	0	0
S2	7	7	0	2
S3	6	6	0	0
S4	8	7	1	0
S5	6	6	0	1
S6	7	7	0	0
S7	6	6	0	0
S8	8	8	0	0
Total	56	55	1	3

Finally, it is important to mention that data of 8 patients used to evaluate the detector (Matched Filter + NN) were not used to build the template of Matched Filter or train the Neural Network.

## Discussion

This paper presents the development of a new mechanism for the automatic detection of epileptic spikes based on the implementation of a neural network built to verify detections made by a matched filter. The matched filter used a template that represents a waveform of an epileptic spike pattern. The tool developed reached a sensitivity of 99.96% and specificity of 99.26% in the identification of epileptic spikes on a dataset with EEG records of children.

The construction of the dataset arose as a need to have a set of training data that describes in detail the beginning and end of an epileptic abnormality, because in the literature there are different datasets that only describe periods of time in which the appearance of an abnormality can be observed and then disorganization or new appearances of the abnormality. One example is the Physionet EEG database [10], which is one of the most widely used pediatric EEG databases. That database does not describe the exact segments of the beginning and end of specific abnormalities.

The main contribution of this work for the field of neurology is the implementation of a method that automatically detects epileptic spikes with high reliability with respect to the values found in the literature. This could decrease the reading time of EEGs and facilitate the diagnosis of Epilepsy by neurologists. Additionally, the proposed method was tested using real data from a Dataset built by the authors and annotated with the help of a neuropediatrician to document the exact segments where the epileptic abnormalities occur in electroencephalograms.

In previous research, many tools have been designed to detect points in EEG signals. The main objective of most of such works is to reduce the reading time of the specialists, since normally they face large volumes of data [11]. In [12] the authors describe the development of a tool for the detection of epileptic spikes using neural networks, in which a PPV (positive prediction value) of 72.67% and a sensitivity of 82.68% were obtained. In [13], an approach is proposed to analyze the EEG record following a Markov paradigm in order to increase the sensitivity of the detection, however, the result becomes a solution with high computational complexity. The work in [14] describes a spike detector developed using analysis of energy and frequency changes; for that, a SNEO (smoothed nonlinear energy operator) is used for testing different window functions; however, the results were reached using a dataset with animal records and the objective of the tool is to support real-time evaluation of EEGs. In [15] a detector of single spikes and spikes with slow waves is proposed. The results of the evaluation show that the model built improves the accuracy of the classification when the single spikes and spikes with slow waves are considered as different classes. The detection is performed in two stages: the first one to detect a possible spike and the second one to extract features of the window and classify it as a spike, a spike with a slow wave or not spike. In that study, the authors performed several configurations, obtaining a sensitivity between 87.9% and 94.4%, as well as a specificity between 86.7% and 92.3%.

Considering the works reviewed, the solution developed in this study obtained a better sensitivity (99.96%) considering the range that has been reported in the literature reviewed (82.68% and 94.4%). Likewise, the specificity reached (99.26%) is better than the specificity of 93% of the best-reviewed work. This was a consequence of the good template built for spikes in brain activity of the children and the threshold used for comparing the autocorrelation matrix of the window with the template, which were obtained empirically.

This proposal showed that the integration of the power of Matched Filter to detect segments similar to a template that describes a spike pattern and to confirm the detected spikes with an additional technique results in a better performance than detectors based on single models. The evaluation carried out demonstrated that when the threshold of Matched Filter was increased, the method not only detected more spikes, but it also slightly misclassified normal segments. In other words, the specificity decreased, and the sensitivity increased. To solve the problem of wrongly detected normal segments, a neural network was trained in order to confirm the detections done by Matched Filter. Thus, two phases considered in the detector allowed us to reach an excellent sensitivity without compromising specificity.

We propose, as future work, the characterization of the greatest number of abnormalities associated with epilepsy in order to develop an epileptic event detector that includes abnormalities other than epileptic spikes. Considering that not all the abnormalities associated with epilepsy can be easily represented in a wave pattern, we also recommend including a classification process based on a process of a general feature extraction through signal processing to support the classification of the segments that cannot be represented through a wave pattern. Finally, the spike detector implemented in this project was tested using EEG records of children; however, this mechanism could be used to detect epileptic spikes in adult patients, since the waveform does not change.

## Conclusion

This paper described the implementation of an epileptic spike detector through the development of a sliding window mechanism that allows the screening of an EEG signal window by window and determining whether or not they correspond to epileptic spikes by comparing a template with each window using a matched filter. The template was constructed from the calculation of the average of 25 segments corresponding to 25 epileptic spikes, and the Matched Filter method implemented achieved a sensitivity of 91.07% and a specificity of 99.96% in the best configuration. However, in order to improve sensitivity, the matched filter was fitted to detect more spikes although this decreased specificity. To solve the problem with specificity, a backpropagation neural network was built to validate the detections made by the matched filter. Thus, the final detector reached a sensitivity of 99.96% and a specificity of 99.26%. Although the results found in this research are preliminary, we consider that the proposal has potential to be applied in a real environment in order to validate and get conclusive results.

The main contribution of this work to the field of neurology is the implementation of a method that automatically detects epileptic spikes with high reliability with respect to the values found in the literature. This could potentially decrease the reading time of EEGs and facilitate the diagnosis of epilepsy by neurologists.

## Data Availability

The datasets generated and/or analyzed during the current study are available in a GitHub repository, https://github.com/Maritzag/EEGSignals.

## Footnotes

1 Segment: for this study, we considered a segment as data extracted from a channel of an EEG.

2 Dataset EEG: https://github.com/Maritzag/EEGSignals.

## References

[1] Garcés A, Orosco L, Diez P, Laciar E (2015). Automatic detection of epileptic seizures in long-term EEG records. Comput Biol Med.

[2] Liliana J, Lara A, Alexandra M, Gómez M, Gómez FR. Informe final Proyecto Estdio de disponibilidad y distribución de la oferta de médicos especialistas, en servicios de alta y mediana complejidad en Colombia. https://www.minsalud.gov.co/salud/Documents/Observatorio%20Talento%20Humano%20en%20Salud/DisponibilidadDistribuciónMdEspecialistasCendex.pdf Accessed 06 Oct 2018

[3] Fraiwan L, Lweesy K, Khasawneh N, Wenz H (2011). Automated sleep stage identification system based on time–frequency analysis of a single EEG channel and random forest classifier. Comput Methods Programs Biomed.

[4] Tsalikakis DG, Tsipouras MG (2017) Epileptic seizures classification based on long-term EEG signal wavelet analysis. In: International Conference on Biomedical and Heatlh Informatics on proceedings Precision Medicine Powered by pHealth and Connected Health, pp 165–169, Thessaloniki, Greece

[5] Acharya UR, Oh SL, Hagiwara Y, Tan JH, Adeli H (2018). Deep convolutional neural network for the automated detection and diagnosis of seizure using EEG signals. Comput Biol Med.

[6] Molina E, López D, Salazar E, NeuroEHR (2018) Open source telehealth system for the management of clinical data, EEG and remote diagnosis of epilepsy. In: 5th Workshop on Engineering Applications on Proceedings, pp 418–430. Springer, Medellín.

[7] Quigg M (2006). EEG pearls: acquisition of the electroencephalogram 2. 1st Edition.

[8] Bancroft JC. Introduction to matched filters, https://crewes.org/ForOurSponsors/ResearchReports/2002/2002-46.pdf Accessed 06 Oct 2018

[9] Hermand J, Roderick WI (1993). Acoustic Model-Based Matched Filter Processing for Fading Time-Dispersive Ocean Channels: theory and Experiment. IEEE J. Ocean. Eng..

[10] Goldberger AL, Amaral LA, Glass L, Hausdorff JM, Ivanov PC, Mark RG, Mietus JE, Moody GB, Peng CK, Stanley HE (2000). PhysioBank, PhysioToolkit, and PhysioNet: components of a new research resource for complex physiologic signals. Circulation..

[11] Gaspard N, Alkawadri R, Farooque P, Goncharova II, Zaveri HP (2014). Clinical neurophysiology automatic detection of prominent interictal spikes in intracranial EEG: validation of an algorithm and relationsip to the seizure onset zone. Clin Neurophysiol.

[12] Carey HJ, Manic M, Arsenovic P (2016) Epileptic spike detection with EEG using artificial neural networks. In: 2016 9th International Conference on Human System Interactions (HSI), pp 89–95

[13] Kumar H, Amit G, Kohli K (2015). EEG spike detection technique using output correlation method : a kalman filtering approach. Circuits Syst Signal Process..

[14] Garg HK, Kohli AK (2013). Nonstationary-epileptic-spike detection algorithm in EEG signal using SNEO. Biomed Eng Lett..

[15] Liu Y, Chou-Ching L, Tsai J-J, Sun Y-N (2013). Model-based spike detection of epileptic EEG data. Sensors.

[16] Elsheikh AH, Sharshir SW, Elaziz MA, Kabeel AE, Guilan W, Haiou Z (2019). Modeling of solar energy systems using artificial neural network: a comprehensive review. Solar Energy..

[17] R. HECHT-NIELSEN (1992) Theory of the backpropagation neural network, no. June 1989. Academic Press, Inc.

[18] Mera-Gaona M, Vargas-Canas R, Lopez DM (2016). Towards a selection mechanism of relevant features for automatic epileptic seizures detection. Stud Health Technol Inform..

